# Duplicated Female Receptacle Organs for Traumatic Insemination in the Tropical Bed Bug *Cimex hemipterus*: Adaptive Variation or Malformation?

**DOI:** 10.1371/journal.pone.0089265

**Published:** 2014-02-19

**Authors:** Yoshitaka Kamimura, Hiroyuki Mitsumoto, Chow-Yang Lee

**Affiliations:** 1 Urban Entomology Laboratory, Vector Control Research Unit, School of Biological Sciences, Universiti Sains Malaysia, Minden, Penang, Malaysia; 2 Department of Biology, Keio University, Yokohama, Kanagawa, Japan; University of Tours, France

## Abstract

During mating, male bed bugs (Cimicidae) pierce the female abdomen to inject sperm using their needle-like genitalia. Females evolved specialized paragenital organs (the spermalege and associated structures) to receive traumatically injected ejaculates. In *Leptocimex duplicatus*, the spermalege is duplicated, but the evolutionary significance of this is unclear. In *Cimex hemipterus* and *C. lectularius*, in which females normally develop a single spermalege on the right side of the abdomen, similar duplication sometimes occurs. Using these aberrant morphs (D-females) of *C. hemipterus*, we tested the hypothesis that both of the duplicated spermaleges are functionally competent. Scars on female abdominal exoskeletons indicated frequent misdirected piercing by male genitalia. However, the piercing sites showed a highly biased distribution towards the right side of the female body. A mating experiment showed that when the normal insemination site (the right-side spermalege) was artificially covered, females remained unfertilized. This was true even when females also had a spermalege on the left side (D-females). This result was attributed to handedness in male mating behavior. Irrespective of the observed disuse of the left-side spermalege by males for insemination, histological examination failed to detect any differences between the right-side and left-side spermaleges. Moreover, an artificial insemination experiment confirmed that spermatozoa injected into the left-side spermalege show apparently normal migration behavior to the female reproductive organs, indicating an evolutionary potential for functionally-competent duplicated spermaleges. We discuss possible mechanisms for the evolutionary maintenance of D-females and propose a plausible route to the functionally-competent duplicated spermaleges observed in *L. duplicatus*.

## Introduction

In several animal groups with internal fertilization, females incur wounds from the male intromittent organ piercing the reproductive tract wall or the body wall, with the wound functioning as an entrance for the male ejaculate. Such a bizarre mode of sperm transfer, termed traumatic insemination (hereafter, TI), is rare but has evolved multiple times independently, with examples from various animal taxa such as insects, spiders, and flatworms [1], [2]. In particular, TI evolved at least three times in the infraorder Cimicomorpha (bed bugs and related true bugs in the order Heteroptera) [2]–[4]. Since Patton and Cragg [5] first revealed that males of the bed bug genus *Cimex* (Cimicidae) stab the abdomen of the female during insemination instead of introducing their copulatory organ into the female vagina, bed bugs (Cimicidae) have been studied as models of TI [1], [2], [6]. Female bed bugs are characterized by a high level of diversity in the paragenital system, which receives the male intromittent organ and ejaculates (e.g., [7]), and this makes bed bugs an excellent study system of the evolution of genital structures.

In the genus *Primicimex,* which is considered to be one of the most primitive representatives among the Cimicidae [6]–[8], females have no special organ to receive sperm. In *Primicimex cavernis* Baber, Carayon [8] found that many females incurred scars scattered across a wide area of the dorsal side of the abdomen, that were indicative of punctures caused by the male intromittent organs (i.e., they were ‘traces of copulation’). In this species, sperm are directly injected into the female hemocoel and then swim to the sperm storage organs, termed seminal conceptacles, that are located at the base of the common oviduct. Thus, insemination is both traumatic and ‘hemocoelic’.

**Figure 1:**
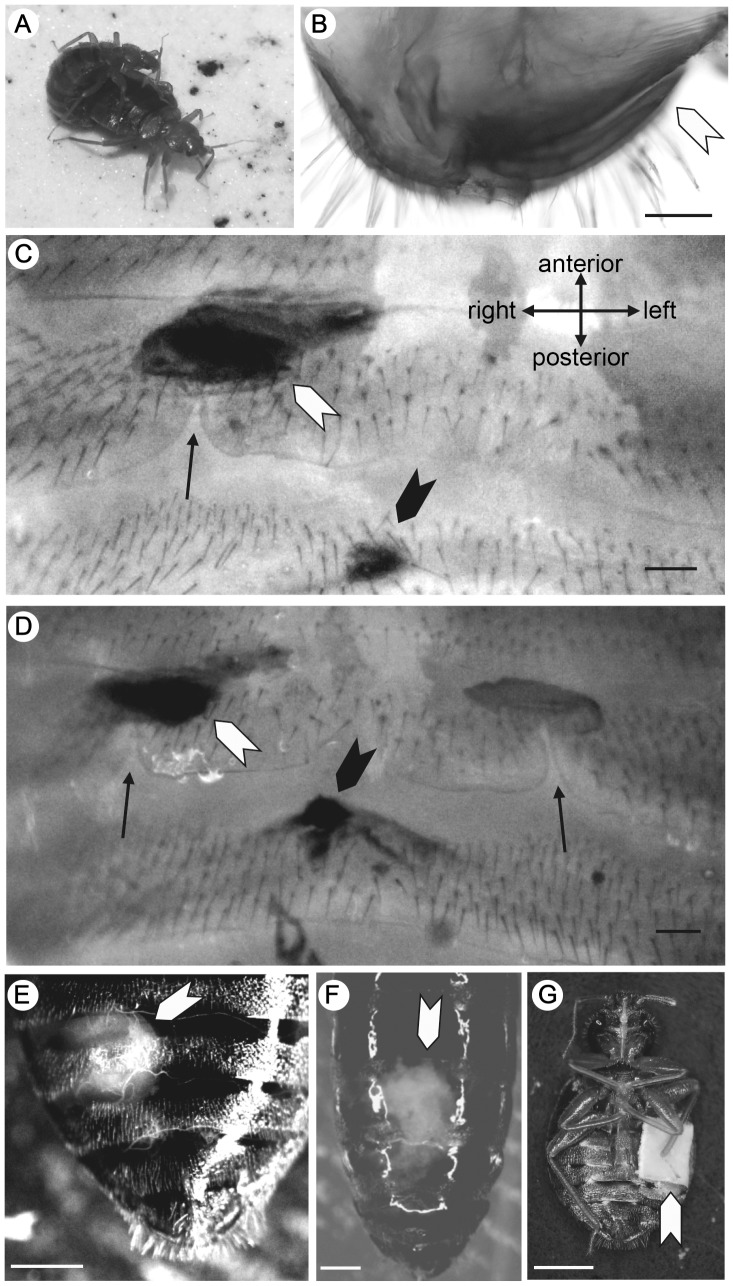
Mating posture (A) and male and female (para)genitalia (B–F) in *Cimex hemipterus*. All pictures except for (A) are ventral views, shown in the direction as indicated in (C). (A) A male mounting a female. (B) Male intromittent organ (paramere) (indicated by the white arrowhead). (C) The paragenital sinus (indicated by the arrow) of an R-female. Cured scars on the exospermalege and in the next sternite are indicated by black and white arrowheads, respectively. (D) The paragenital sinuses (indicated by the arrows) of a D-female. Scars are indicated as in (C). (E) An R-female at 40 min after a normal copulation. Sperm in the mesospermalege (the white arrowhead) can be seen through the thin sternites. (F) An accidentally discovered R-female with an injected sperm mass outside the spermalege (indicated by the white arrow head). Unlike normally inseminated females, the sperm mass showed no sign of migration even 10 hours after discovery. (G) An example of females with the left-side abdomen sealed. See text for the details about the experiment. Scale bars, 100 µm in C–D, and 1 mm in E–G.

**Figure 2:**
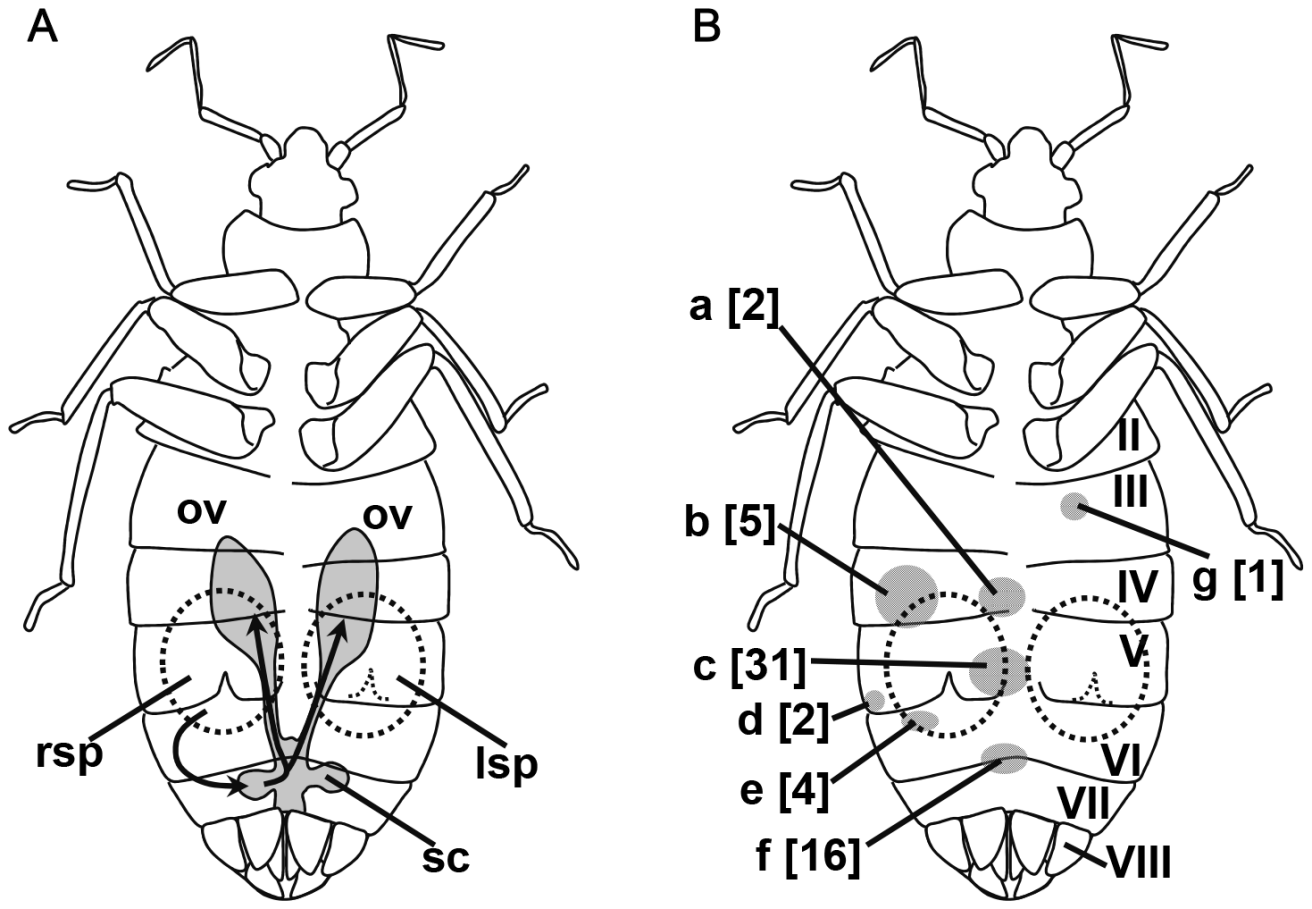
(Para)genital systems and the distribution of cured scars on the abdomen of female *Cimex hemipterus*. (A) A schematic drawing of the genital and paragenital systems. The migration routes of sperm are indicated by the arrows. The migration routes of sperm are indicated by the arrows. (B) Distribution of cured scars on the female abdominal exoskeletons outside the spermalege(s). The roman numerals (II-VIII) indicate the 2nd-8th abdominal sternites, respectively. Sites a, c, d, and f are on inter-segmental membranous regions, whereas b, e, and g are on hardly screlotized parts of the sternites. The numbers in the brackets indicate the number of females with scar patches on each site. Abbreviations: lsp, left mesospermalege; ov, ovary; rsp, right mesospermalege; sc, seminal conceptacle.

Two species of the genus *Cimex*, *C. lectularius* Linnaeus and *C. hemipterus* (Fabricius), are the most well-studied representatives of bed bugs. *Cimex* species show an intermediate state of specialization for TI [7]. Females possess a spermalege, an organ specialized to receive hypodermically injected semen, on the right side of their abdomen. The spermalege consists of two parts; the ectospermalege, a modification of the exoskeleton with an ectodermal origin, and the mesospermalege, an internal organ with a mesodermal origin [7]. In *Cimex* spp., males pierce the female abdomen at a slit-like sinus of the ectospermalege at the posterior edge of the 5th abdominal sternite (e.g., [5], [7]; Fig. 1C, D) with their needle-like genitalia (the paramere: [9]). At the base of the sinus, the paramere penetrates a thickly sclerotized ectodermal wall and injects semen into the mesospermalege, which is a soft spherical bag. The spermatozoa then form a mass and diffuse through the wall of the mesospermalege, swimming through the hemocoel to a pair of seminal conceptacles, where sperm are stored before they migrate to the ovaries for fertilization ( [7]; Fig. 2A). Thus, although females of *Cimex* have a specialized sperm receptacle organ and injection of sperm is not directly ‘hemocoelic’, there is a phase in which sperm are in direct contact with the female hemolymph. A similar condition was reported for other genera of Cimicidae (e.g., *Afrocimex*; [7]). In a supposedly most advanced stage of the evolution of paragenital systems, females of *Stricticimex brevispinosus* Usinger and *Crassicimex* spp. (*C. sexualis* Ferris & Usinger and *C. pilosus* Ferris) possess a specialized duct, termed the conductor cord, that directly connects the mesospermalege and the base of the common oviduct [7], [10]. Sperm injected into the mesospermalege travel to the genital tract through the cord, and thus insemination is still traumatic but not hemocoelic in these genera. Based on the hypothetical relationships of cimicids [6], these various states enable us to estimate the history of male-female coevolution related to TI, during which gradual specialization of the female paragenital systems (spermaleges and associated structures) was accompanied by localization of insemination sites and separation of injected ejaculates from the female hemolymph.

Another interesting variation observed in Cimicidae is duplication of the paragenital system in females of *Leptocimex duplicatus* Usinger. Carayon [7] found that both of the independent paragenitalia, which are located on each side of the abdomen, are used to receive ejaculates. In contrast, females of other species of this genus have a single spermalege on the right side of the abdomen [7]. The significance and origin of this evolutionary novelty are unknown. Interestingly, in *C. hemipterus*, *C. lectularius*, and *Oeciacus hirundinis* (Lamarck), in which normal females develop a spermalege only on the right side, similar duplication of the paragenital system has been sporadically observed and reported [11]–[14]. Cragg [11] first reported two examples of females with two spermaleges, one on each side of the abdomen, in his *C. lectularius* population. Following the terminology of Ludwig [13], females with duplicated spermaleges are hereafter called D-females, whereas normal females with a spermalege only on the right side are referred to as R-females. Considering sample numbers from previous studies, Cragg [11] estimated the proportion of D-females to be less than one per several hundred R-females. However, Abraham [12] found a high variation in the proportion of D-females (2.6–37.9%) among his three populations of *C. lectularius*. He failed to detect sperm in the left-side spermaleges of D-females, although the number of samples examined is unclear. Ludwig [13] examined multiple strains of *Cimex* spp. and found that D-females also occur in *C. hemipterus* (referred to as *C. rotundatus* in Ludwig 1937; 0–16.5%; 2 strains examined) as well as *C. lectularius* (0–7.7%; 13 strains examined). To date, no comprehensive study has been conducted to evaluate the function of the duplicated sperm-receiving organs in females of *Cimex*. Such a study, however, might help explain the evolutionary novelty of duplicated spermaleges being the norm in *L. duplicatus.*

We propose two possibilities to explain their occurrence. First, the left-side spermalege of *Cimex* spp. may not function as an organ for receiving ejaculates. In this case, D-females merely represent malformations. Duplication of a normally asymmetrical structure results in a laterally symmetrical body plan in malformed individuals. Such aberrations, termed symmetrical repeats, are usually very rare (see Discussion). Thus, the high frequencies of occurrence, which sometimes reach nearly 40% in *C. hemipterus* [12], warrant elucidation of their evolutionary maintenance. Second, the left-side spermaleges may function as a safety-net for misdirected piercing by male genitalia and possible subsequent ejaculation. Piercing of a female body outside the spermalege is rare but has been reported for *Cimex* spp. and other bed bug species (e.g., [12], [14], [15]; see also Fig. 1E–F). Using an artificial insemination (AI) technique, Davis [16] clearly showed that the spermalege minimizes the detrimental effects associated with TI. In *Cimex*, seminal fluid components are necessary for the spermatozoa to acquire fertilization competency [16], [17]. This activation process takes place in the mesospermalege, where spermatozoa from the testes and seminal fluids from the accessory glands are deposited in a mixture [16]. Davis [16] reported that injection of free sperm into the hemocoel before activation resulted in high female mortality (31.6%), whereas no female died during the 1-week observation period when free sperm were injected into the spermalege. Moreover, even after activation, injection of a normal amount of semen (i.e., the volume of a single ejaculate: ca. 0.6 µl) directly into the hemocoel was usually fatal; even with a reduction in the volume of injection to 0.2 µl, mortality was 45.0% and the oviposition rate was 65.0% [16]. Because the mesospermalege contains a large number of hemocytes (e.g., [12]), which may attack and consume sperm [7], [18], their immunological function likely explains the observed mitigation of the deleterious effects of injected spermatozoa, especially those that are not activated by seminal fluids. In a pair of similar experiments, Morrow and Arnqvist [19] and Reinhardt et al. [20] artificially pierced both the spermalege and the corresponding site on the left-side abdomen of female *C. lectularius* using sterilized and non-sterilized needles. Effects of the simulated mating trauma revealed cost-mitigating functions of the spermalege against the mating costs associated with the trauma itself and bacterial contamination. Thus, hemocytes in the spermalege are also considered to be an adaptation to counter pathogens carried on the male genitalia [21]. It is possible that the left-side spermalege of D-females functions as an adaptation to counter misdirected genital piercing by males. Interestingly, male bed bugs cannot discriminate sex before genital contact [22], resulting in lethal homosexual insemination on some occasions [23], [24]. The costs of TI mentioned above, and accidental rupture of the unprotected gut [25], can explain the fatal effects of homosexual copulation. In *Afrocimex constrictus* Ferris and Usinger, males also develop paragenital sinuses, which conspecific males will pierce during misdirected mating attempts [26]. The male form of the paragenitalia differs morphologically from the standard female form. Moreover, some females have the male-like paragenitalia and experience reduced copulatory wounding, suggesting sexual mimicry as a counteradaptation to sexual conflict over an optimal mating rate [26]. Theories predict that sexually antagonistic coevolution driven by sexual conflict sometimes results in a dimorphism in females [27]. The intraspecific diversity in *Afrocimex* and *Cimex* may represent different female strategies caused by sexual conflict.

During the course of our study of the resurgence of the tropical bed bug, *C. hemipterus* in Southeast Asia [28]–[30], we discovered dimorphic variation in the female spermalege in our laboratory-maintained colony. In this study, based on detailed examination of abdominal exoskeletons of females, we show that males sometimes pierce various sites outside the spermalege. By using experimental manipulation of the normal piercing site and AI techniques, the present study addresses the question of whether the left-side spermaleges of D-females are functional duplicates or non-functional malformations.

## Methods and Techniques

### Insects, Rearing and Morphological Observations

We used a laboratory stock population of *C. hemipterus* that was established from insects collected in Serangoon, Singapore in 2005 [31]. Colony maintenance followed the rearing protocol of How and Lee [28]–[30]. The population was maintained in a glass jar (7 cm diameter, 9 cm height) at 26±2°C and 70±5% relative humidity with folded brown paper as a harborage and oviposition site. Bed bugs were fed on a human volunteer (who is the senior author of this paper) at 10 day intervals. Wattal and Kalra [32] reported that humans are the preferred host of *C. hemipterus* among bulbuls, chickens, rabbits and rats. Virgin adults at 3–5 days old were used for the mating experiments described below. To collect virgin adults, we separated final-instar nymphs individually in 1.5 ml plastic centrifuge tubes containing a small piece of folded brown paper. Emergence to adults was checked every 3 days.

For histological comparisons between the right-side mesospermalege (of both R- and D-females) and the left-side ones (of D-females), mesospermaleges were dissected out in insect Ringer’s solution (0.9 g NaCl, 0.02 g CaCl_2_, 0.02 g KCl, and 0.02 g NaHCO_3_ in 100 ml water) from freshly killed females. The samples were fixed in 20% formalin for 1–3 days and then preserved in 70% ethanol for later examination. The samples were dehydrated in a graded ethanol-butanol series and embedded in paraffin. Serial sections 5 µm in thickness were stained with Mayer’s haematoxylin and eosin. Because of the scarcity of D-females, several females used in the following experiments (the sealing and artificial-insemination experiments) were later used for the histological examination.

### Distribution of Copulatory Wounds

Dried dead bodies of both sexes were collected from the culture bottle. To digest their internal organs, we boiled the female specimens in 20% NaOH solution for two hours. Treatment in alkaline solutions dissolves soft tissues (including mesospermaleges and the mass of scar substances on the soft tissues) but leaves exoskeletons and scars (i.e., traces of copulation) on them [7]. The digested content was carefully removed by making a small hole in the intersegmental membrane between the metathoracic and first abdominal tergite using fine forceps. The exoskeleton samples were then carefully washed in water and observed from the ventral side under an SZ61 stereo microscope (Olympus, Tokyo, Japan). Digital images of the samples were obtained using a X-cam α CCD camera and Digi-Acquis software (Matrix Optics, Selangor, Malaysia). As a control for this observation, the exoskeleton of virgin females (*n* = 20) was observed by the same method.

Aberrant males with duplicated intromittent organs have been reported for *C. lectularius* (only two examples out of 3575 males examined; [13]) and *C. hemipterus* ( [7]; with unknown frequency). These males (D-males) were considered to be sterile [7], [13]. In the current study, dead male bodies were also examined under the stereo microscope to check for the occurrence of D-males.

### Spermalege Sealing Experiment

We conducted the following experiment to determine whether males can inseminate females by piercing a location other than the normal spermalege located on the right side of the abdomen. The site of normal insemination and nearby areas of virgin females (sternites II–V) were covered by a 1×1 mm piece of paper (Power Pine Seal label, No. 2470, Well Stationery Mart Sdn. Bhd., Shah Alam, Malaysia; Fig. 1G) using a drop of instant adhesive (Aron Alfa Superglue-jelly type, Toagosei Co. Ltd., Tokyo, Japan). For this treatment, the females were lightly anaesthetized by placing them on an ice-chilled plaster board with their ventral side up. One day after the sealing treatment, the females were fed human blood *ad libitum*, and then they cohabitated with a randomly chosen, virgin, fully fed male for 9 days in a 1.5 ml microcentrifuge tube containing a small piece (15×15 mm) of folded brown paper as a resting and oviposition site.

Three types of control were prepared for this experiment: a virgin control, an experimental control, and a positive control. The negative controls were virgin females kept without a male, whereas the positive controls were kept with a virgin male. The females in the experimental control group were treated the same as the treatment group, but the left side of the ventral surface of the abdomen was sealed (Fig. 1G). After 9 days, the number of eggs laid was recorded for each female. When the pigmented compound eyes of the embryo were detected through the egg shell in at least one egg, the females were scored as inseminated. For R-females, we prepared 20 replicates for each of the four groups. However, because of the rarity of D-females, we conducted a similar experiment for D-females using three females in the treatment group and two females in the positive control group. No females died during the observation period. Three samples (two and one cases in the treatment group and experimental control of R-females, respectively) were discarded from the subsequent analysis because the sealing paper dropped off during the observation period.

### Artificial Insemination Experiment

To determine whether the left-side spermalege of D-females is functionally competent, we conducted an AI experiment. Davis [16] noted three difficulties for AI experiments in *C. lectularius*: (1) injection of only sperm without seminal fluid directly into the hemolymph causes high mortality compared with injection into the mesospermalege; (2) hemocoelic injection of ejaculate in the amount ordinarily transferred by males is usually fatal; and (3) sperm motility depends greatly on the saline used to dilute them. To avoid these problems, ejaculate that was freshly deposited into the mesospermalege of a female via normal copulation was sampled and immediately injected into the mesospermalege of a virgin female (treatment, *n* = 3 for the left-side spermalege of D-females; control, *n* = 6 for the right-side spermalege of R-females). First, a recipient female was immobilized on a glass slide with a small piece of double-sided adhesive tape (12×15 mm, 1 mm thickness; PV-2, Sumitomo-3M, Tokyo, Japan). Males and virgin R-females were allowed to mate. A mated female (the donor) then was immobilized on the slide immediately after completion of normal copulation. A puncture was made in the ectospermalege of both the donor and recipient using the tip of fine insect pin (diameter, up to 50 µm). The ejaculate that was deposited in the mesospermalege of the donor was removed and immediately injected into the mesospermalege of the recipient using a simple, mouth-operated aspirator. The aspirator consisted of a fine plastic needle with a tapered tip (inner diameter, ca. 75 µm; outer diameter, ca. 200 µm), a Tygon connection tube (3.2 mm diameter, 450 mm long), and a mouthpiece. To estimate the amount of artificially injected ejaculate, we injected ejaculates into mineral oil using the same method as the AI manipulation. Based on the diameter of spherical ejaculate drops, the volume was estimated to be 0.065±0.036 µl. The recipients were kept individually in the rearing tube for 5 hours, and then lightly anaesthetized in a freezer. The mesospermalege and the seminal conceptacles were dissected out of the samples in insect Ringer’s solution under the stereomicroscope, and examined under a light microscopes (BX53, Olympus; 40–400×). A pilot experiment revealed that at 5 hours after normal copulation, sperm evacuating the mesospermalege and sperm reaching the seminal conceptacles can be observed simultaneously.

### Statistical Analysis

We used a Fisher’s exact test to analyze count data and standard parametric tests for variables that conformed to the assumptions of homogeneity of variances and a normal distribution. All tests were two-tailed, and acceptance levels were adjusted using the sequential Bonferroni procedure [33] to α = 0.05. All statistical analyses were performed using R version 3.0.1 [34]. Data are presented as means ± one standard deviation unless otherwise stated.

## Results

### Variation in Genitalia and Misdirected Mating Wounds

**Figure 3:**
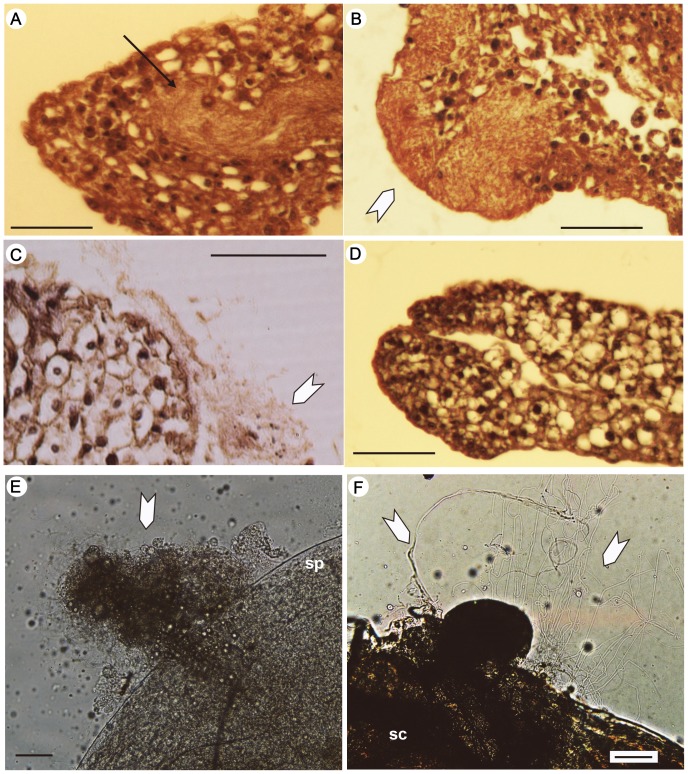
Cross-sections of the mesospermalege and the artificially injected spermatozoa in female *Cimex hemipterus*. (A–D) Cross-sections of the left-side mesospermalege of a D-female (A, B), and the right-side mesospermalege of a D-female (C) and an R-female (D). Sperm injected by AI are indicated by the arrow (A). The white arrowheads in B and C indicate a sperm mass evacuating the mesospermalege. (E) A sperm mass (indicated by the white arrowhead) evacuating the left mesospermalege of a D-female 5 hour after the AI treatments. (F) Spermatozoa (indicated by the white arrowheads) entering the sperm conseptacles of a D-female at 5 hour after the AI treatments. Abbreviations: sc, seminal conceptacle; sp, mesospermalege. All scale bars, 50 µm.

Although rare, we found a total of 15 D-females, which had the paragenital sinuses on both the right and left sides of the 5th abdominal sternite (Fig. 1D). Ludwig and Zwanzig [14] reported variation in the development of the left-side spermalege that ranged from morphologically complete copies of the right-side ones to only a paragenital sinus (ectospermalege) without the mesodermal components in D-females of *C. lectularius*. In our samples, all of the D-females had a well-developed left-side mesospermalege, except for the dead body samples described below for which we could not examine the mesodermal components. The left-side mesospermaleges of D-females were filled with many cells and showed no detectable differences in the histology from the right-side mesospermaleges (of R- and D-females; Fig. 3A–D). Based on the morphology (e.g., [18]), these cells were identified as hemocytes, which are responsible for the immunological function of the mesospermalege.

Among the 185 dead female bodies examined, 179 were normal R-females and the other 6 (3.24%) were D-females. In *C. hemipteru*s, the superficial cuticle of the ectospermalege forms a palisade-shaped structure, which is diagnostic in virgin females after the alkaline treatment ( [7]; see the right side of Fig. 1D). This previous observation was confirmed for our virgin control samples. One hundred eighty-two females (177 R-females and 5 D-females) had at least one blackish or dark brownish patch on this structure of the right-side ectospermalege, indicating that they had mated at least once using the right-side spermalege (Fig. 1C, D). The other three females (two R-females and one D-female), which showed no sign of scars on the spermalege(s), were considered to have died as virgins.

Detailed examination of the abdominal sternites revealed that 28.1% (52 out of 185) of the females had at least one scar outside the ectospermalege(s) (Fig. 2B). The number of scarred sites ranged from one to three (1.17±0.43). Except for a single case (site g in Fig. 2B), those scars were found on the right side of the female abdomen, especially in the intersegmental membranous regions of the 4th–6th sternites near the right spermalege. This highly right-biased distribution of scars (the number of scar patches detected, right:left = 61∶1; binomial test, *P*<0.0001) suggests that they were caused by piercing by the male paramere during normal copulation attempts, which are highly biased toward the right side of the female abdomen as discussed below. Among the six D-females, scars located outside of the right spermalege were detected in three females (including one possible virgin). The occurrence rate of scars outside the right spermalege did not significantly differ between the R- and D-females (Fisher’s exact test; *P* = 0.35). No sign of mating was detected on the left-side spermaleges of the D-females.

In the 183 dead males examined, no abnormality was detected with respect to the laterality of the male genitalia. In all males examined, a single paramere directed towards the left side of the body was found (Fig. 1B). The occurrence rate of lateral abnormalities (genital or paragenital duplication) was significantly higher in females than in males (Fisher’s exact test; *P* = 0.03).

### Spermalege Sealing Experiment

**Figure 4:**
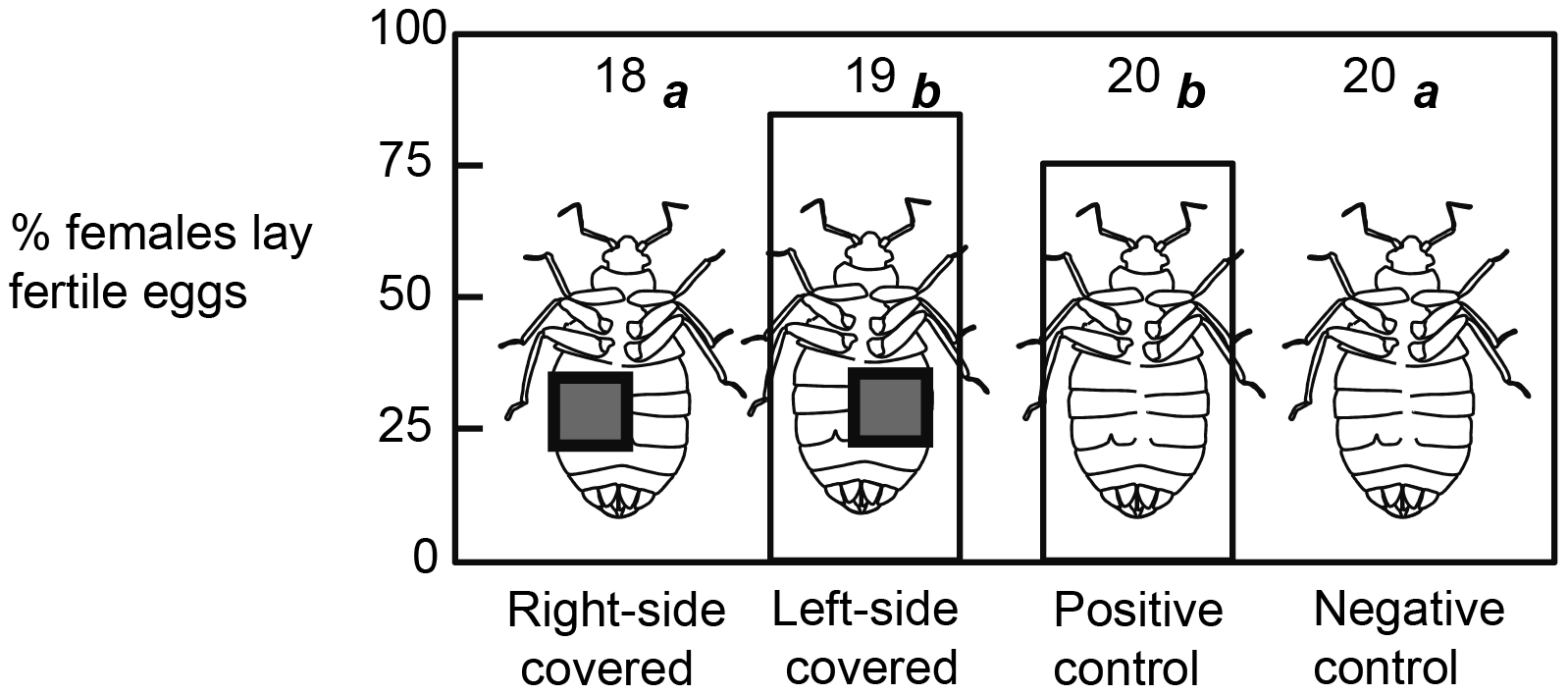
Proportion of female *Cimex hemipterus* that laid fertile eggs in the sealing experiment. The numbers above the columns indicate the sample sizes. A significant difference was detected between bars labeled with *a* and *b* (Fisher’s exact test, *P*<0.0001 after a sequential Bonferroni method), whereas bars labeled with the same letters were not significantly different (*P*>0.05).

When the left side of the mid abdomen was covered with a square piece of paper, 84.2% of the treated R-females produced at least one fertile egg. This rate was not significantly different from that of the positive control (Fig. 4). There was no significant difference in the number of eggs laid during the 9-day observation period between these two groups of females (3.42±2.60 for the experimental control, 2.95±2.35 for the positive control; *t*-test, *t*_37_ = 0.663, *P* = 0.51). In contrast, no female laid eggs when the right side of the abdomen was covered or when the female was kept without a male (Fig. 4). Although statistical tests could not be conducted because of the small sample size, the two D-females in the positive control laid four and seven eggs, respectively, during the observation period. In contrast, all three D-females of the treatment groups remained infertile as was the case with the R-females.

### Artificial Insemination Experiment

At 5 hours after AI, successful migration of sperm was observed in all the treated females (right-side spermalege of six R-females and left-side one of three D-females; Fig. 3B, C, E, F). As in the case of normally mated females, vigorous flagellation was observed in spermatozoa forming the sperm mass (Fig. 3B, C, E), and a small amount of motile sperm was also detected at the seminal conceptacles in all females examined (Fig. 3F).

## Discussion

Irrespective of the similarity in the histology and sperm-evacuation process observed in the AI experiment between the normal right-side spermaleges and duplicated left-side ones, we detected no sign that the latter is used by males for insemination. It is noteworthy that the left-side spermaleges were never used for insemination even in the cases in which the right ventral side of the abdomen was artificially covered. Thus, D-females observed in *C. hemipterus* are likely to be malformations rather than a form of adaptive polymorphism. One possible cause of disuse of the left-side spermaleges is the asymmetry in the male genitalia, which always points to the left side (Fig. 1B). As shown in this study and by Ludwig [13], malformations in male genital asymmetry are much rarer than in females, suggesting that different genetic mechanisms control the asymmetry in males and females. However, the left-directed paramere (at least at the proximal part) is a characteristic shared by other members of the Cimicidae, including *Afrocimex*, in which females have a single spermalege on the left side [7], [26], and *L. duplicatus* in which females have paired spermaleges [7]. For a pair of sympatric sister species of *Coridromius* (Miridae), Tatarnic and Cassis [35] recently reported that female *C. tahitiensis* has a spermalege on the right side of the abdomen, whereas *C. taravao* likely receives TI on the left side, despite the lack of differentiation in the laterality of male genitalia. Interestingly, costs of interspecific TI are suggested to have caused the evolutionary switching. Instead of changes in morphologies, lateralized male behavior (handedness) is a more plausible cause of the laterally biased distribution of scars and observed disuse of the left-side spermalege in *C. hemipterus*. When a male of *Cimex* mounts a female, he always curls his abdomen to contact with the right ventral side of the female abdomen from the right side (Fig. 1A). Males show this unidirectional mating posture even without any mating experience ( [36], YK, pers. obs.). Siva-Jothy [37] found that females of *C. lectularius* who were unwilling to mate exhibited a refusal posture, in which they pressed the right ventral side flat to the ground to make their right-side spermalege inaccessible to males. Given that male mounting behavior is highly lateralized, this female behavior can be considered to be an effective counteradaptation to harassment by males.

Specifically restricting piercing to the spermalege is important for female survivorship. Male animals sometimes evolve a specialized morphology or behavior that damages female mates when the associated benefits outweigh the costs. A well-studied example is the evolution of seminal fluid chemicals in *Drosophila* fruit flies that increase the immediate oviposition rate and unreceptivity to remating while reducing female longevity and lifetime fecundity (e.g., [38]). Such sexual conflict over an optimal mating rate also has been reported for *C. lectularius* [39]. However, in *Cimex* spp., the cost of ejaculation directly into the female hemocoel is likely to be too costly to females (and thus also to males) to function as a viable mating tactic against unwilling females. Females that receive ejaculate directly into the hemocoel suffer both reduced survival and reduced fertilization success [16]. Male *C. lectularius* evolved another mating tactic: Males preferentially mate with fully fed females with reduced resistance [40]. Previous researchers assumed that TI of Cimicidae had originated from misdirected hemocoelic insemination in an ancestor that normally used the reproductive tract orifice to receive ejaculate (e.g., [15]). If such misdirected hemocoelic insemination was beneficial for males as a means to bypass pre- and/or postcopulatory female resistance, TI was likely to evolve [1], [2]. However, it is obvious that from a possible *Primicimex*-like ancestor, in which males pierced unspecified sites of the female abdomen for insemination, females of *Cimex* likely lost their ability to cope with sperm directly injected into the hemocoel [16]. Such reduced fertilization success of extra-spermalege insemination may have played an important role in the correlated evolution of male mating behavior and female paragenital morphology.

In this study, we did not detect a function for the spermaleges located on the left side of the female abdomen. However, if D-females represent merely malformations, the observed frequency seems much higher than that of malformations of other laterally asymmetrical structures observed in other animals [13], [41]. For animals with a normally asymmetrical body plan, malformations in the laterality can be divided into two major categories: symmetrical repeats and reversals [41]. The former type (i.e., secondary symmetry by duplication of a laterally asymmetrical structure) is rare but has been reported for genital structures of several other insects (e.g., [42]–[45]). However, Ludwig [41], who compiled many examples of variations in lateral asymmetries, concluded that symmetrical repeats are generally much more rare than reversals for animal structures showing directional asymmetry. This is in striking contrast to female *Cimex*, for which duplications are 30 times more frequent than reversals [13], and it is especially enigmatic considering that the spermalege occupies a considerable space in the female abdomen (see Fig. 2A, B). Although the abdomens of bed bugs are highly expandable [40], the spermaleges are likely to compete for space with the ovaries and the gut, which functions as a food reservoir in this sit-and-wait predatory insect. Crossing experiments conducted by Ludwig [13] failed to detect a simple genetic basis for the production of D-females, as the probability of producing D-females was not different between R- and D-female mothers. Instead, inter-crossing among strains with varying D-female frequencies clearly revealed that the propensity for producing D-female offspring has a genetic basis that is free from maternal or cytoplasmic factors [13]. Therefore, the frequency of D-females is an evolvable trait and can respond to selection pressures. One possible explanation for the evolutionary maintenance of D-females is that they represent atavisms to ancestors that possessed paired spermaleges. Although a completely symmetrical (paired or unpaired) spermalege is rare among extant cimicids [7], such hypothetical ancestors can explain D-females as individuals caused by recurrent back mutations. Alternatively, formation of a large organ in only one side of the body may be ‘a developmentally difficult task.’ Specifically, given that reversals are likely to be sterile or fatal in female *Cimex*, molecular mechanisms underlying the development of the spermalege are likely to have been canalized not to produce reversals, and such mechanisms may incline to produce duplications as a result of a genetic constraint. To test this hypothesis, the developmental mechanisms of spermaleges and the genes controlling them must be examined in the future.

Can *L. duplicatus* evolve from a *Cimex*-like ancestor? The discussion above sheds light on one plausible route to the evolution of functionally-competent duplicated spermaleges. Let us consider a mutation that changes the laterality of male mating attempt behavior. The mutant male can attempt to mate females from both their right and left side. This male may have an advantage over others if females hide the right-side spermalege by exhibiting the refusal posture [37]. This advantage must be dependent on the frequency of D-females: If D-females are frequent, the mutant can exclusively use the left-side spermalege, whereas piercing the left-side of R-females reduces the probability of fixation of this mutant, given that ejaculates are not a limitless resource [46]. High frequencies of D-females are likely to select for non-lateralized male behavior, which may in turn select against R-females. Thus, given that D-females are sometimes highly frequent (up to 37.9%; [12]) and that the occurrence rate of D-females has a genetic basis, a small change in male behavior could result in the evolution of the functionally-competent duplicated spermaleges. To test this hypothesis, detailed comparative studies of the ecology and ethology of *Leptocimex* spp. are warranted.

## Acknowledgments

This study was conducted with the approval of the Economic Planning Unit, Malaysia (Reference No. UPE: 40/200/19/2844). We thank F.-K. Foo for assistance with the rearing of the bedbug colony, H.-S. Tee for providing important references, and L.-H. Ang and M.-K. Kuah for their invaluable advice about the experimental equipment. We are also grateful to N. J. Tatarnic for his helpful comments on an earlier version of the manuscript.
